# Identification and characterization of a neutralizing monoclonal antibody that provides complete protection against *Yersinia pestis*

**DOI:** 10.1371/journal.pone.0177012

**Published:** 2017-05-09

**Authors:** Weicen Liu, Jun Ren, Jinlong Zhang, Xiaohong Song, Shuling Liu, Xiangyang Chi, Yi Chen, Zhonghua Wen, Jianmin Li, Wei Chen

**Affiliations:** Laboratory of Vaccine and Antibody Engineering, Beijing Institute of Biotechnology, 20 FengTai Dongdajie Street, Beijing, PR China; Shanghai Medical College, Fudan University, CHINA

## Abstract

*Yersinia pestis (Y*. *pestis)* has caused an alarming number of deaths throughout recorded human history, and novel prophylactics and therapeutics are necessary given its potential as a bioweapon. Only one monoclonal antibody has been identified to date that provides complete protection against *Y*. *pestis*. Here, we describe a second novel murine monoclonal antibody (F2H5) that provided complete protection against *Y*. *pestis* 141 infection when administered prophylactically to Balb/c mice (100 μg intravenously). We humanized F2H5, characterized its ability to bind to the *Y*. *pestis* F1 protein and further characterized the neutralizing epitope using computational and experimental approaches. While Western blot results suggested a linear epitope, peptide mapping using ELISA failed to identify an epitope, suggesting a conformational epitope instead. We adopted a computational approach based on Residue Contact Frequency to predict the site of antigen-antibody interaction and defined the F2H5/F1 binding site computationally. Based on computational approach, we determined that residues G^104^E^105^N^106^ in F1 were critical to F2H5 binding and that CDRH2 and CDRH3 of F2H5 interacted with F1. Our results show that combining computational approach and experimental approach can effectively identify epitopes.

## Introduction

*Yersinia pestis* (*Y*. *pestis*) is the causative agent of the plague, which has killed an estimated 160 million people throughout recorded history [1]. *Y*. *pestis* is difficult to eradicate because animal reservoirs exist worldwide. According to a World Health Organization (WHO) report, between January 2010 and December 2015, there were 3,248 cases of *Y*. *pestis* infection worldwide with a mortality rate of 17.98% [2]. *Y*. *pestis* also has the potential for using as an aerosolized bioweapon and is recognized as a category A agent on the National Institute of Allergy and Infectious Diseases (NIAID) list of biodefense-related pathogens [3].

The first line antibiotics for treatment of *Y*. *pestis* are streptomycin, tetracycline, and chloramphenicol, while the first line prophylactics are sulfonamide, trimethoprim-sulfamethoxazole, or tetracycline. A strain of *Y*. *pestis* with resistance to all of the antimicrobial agents recommended for treatment and prophylaxis was isolated in 1995 in Madagascar from a 16-year-old male presenting with symptoms of bubonic plague. The isolate’s drug resistance was mediated by a self-transferable plasmid, raising the potential for wider dissemination and a possible threat to global public health [4]. The former Soviet Union developed a live attenuated vaccine against *Y*. *pestis* that prevented infection, but did not have therapeutic efficacy [5]. Monoclonal antibodies (mAbs), such as PAmAb and ETIi204 targeting *Bacillus anthracis*, might be an alternative therapeutic when common therapies are not available or appropriate [6,7].

Vaccine development for *Y*. *pestis* has focused on the Fraction 1 Capsular Antigen (F1) [8–10]. The low-calcium-response V antigen (LcrV) and other antigens have been investigated as vaccine targets [11–13], but the results were not promising. In murine models, three mAbs against F1, F1-04-A-G1, F1-08-D-G1 and YPF1-6H3-1-1, have protected 60%-100% of mice challenged subcutaneously with *Y*. *pestis* [14]. In addition, a human F1 specific mAb (M252) has been isolated that results in approximately 33% survival in an in vivo *Y*. *pestis* challenge model [15]. To date only, F1-04-A-G1 has shown to provide complete protection. These results suggest that there is at least one critical neutralizing epitope in the F1 protein. However, the number of protective epitopes in the F1 protein is not yet known and the epitope recognized by F1-04-A-G1 has not been reported. M252 has been reported to bind weakly to the immunodominant peptide in F1 (amino acids 142–165), but unfortunately, this epitope is not neutralizing [15].

Here, we describe a mAb (F2H5) from a mouse hybridoma that provides complete protection in a mouse *Y*. *pestis* infection model. We also characterized the binding epitope using computational algorithms for predicting complex structures and binding sites when experimental approaches failed. By this method, we identify the epitope successfully.

## Materials and methods

### Ethics statement

All the animal experiments in this study were approved by the Laboratory Animal Care and Use Committee of Beijing Institute of Biotechnology. All surgery was performed under sodium pentobarbital anesthesia and mice were sacrificed at indicated time by CO2 inhalation. All efforts were made to minimize the suffering.

### Cultivation of virulent *Y*. *pestis*

A virulent strain of *Y*. *pestis* (141) was isolated from *Marmota himalayana* on the Qinghai-Tibet plateau by Qinghai Institute for Endemic Disease Prevention and Control [16]. *Y*. *pestis* 141 (Sample ID: 11001) has a median lethal dose (MLD) of 17 colony-forming unit (CFU) when subcutaneously administered to BALB/c mice [17]. *Y*. *pestis* was cultured in Luria-Bertani (LB) broth at 28°C for 18 h then quantified by Maxwell turbidimetry and diluted in sterile phosphate-buffered saline (PBS). The number of *Y*. *pestis* in the dilution was verified by colony-forming units (CFU) on *Y*. *pestis* selective agar medium.

### Expression of wild type and mutant F1 proteins

Expression and purification of recombinant F1 (rF1) has been described previously [17]. Briefly, the F1 gene was cloned into the expression vector pET-32a (+) to construct the final vector pET-F1, which was transformed into BL21(DE3) cells to obtain BL21(DE3)/pET-F1. The BL21 (DE3)/pET-F1 cells were grown in LB broth until the OD_600_ reached 0.6. Protein expression was induced using isopropyl-beta-D- thiogalactopyranoside (IPTG) at a final concentration of 1 mM for 5 h. The pellets were collected by centrifugation and then homogenized by ultrasonication. Following centrifugation, the soluble extract was decanted from the insoluble pellet fraction. The rF1-containing pellet was extracted in 25 mL of extraction buffer (8 M urea, 20mM PB, pH 6.0) with vigorous mixing for 15 min at room temperature. The extraction procedure was repeated four times. A total of 100 mL of rF1-containing urea extract was obtained per liter of cultured BL21 (DE3)/pET-f1. The urea was dialyzed out of the extraction buffer in a stepwise fashion (urea concentrations:4M, 2M, 1M, 0.5M, 0M) until rF1 was resuspended in PBS without urea. The same procedure was followed for the rF1 mutants.

### Monoclonal antibody generation and in vivo efficacy testing

Balb/c mice were immunized with rF1 to prepare monoclonal antibodies using conventional hybridoma methods [18]. Four monoclonal antibody lines named F2H5, F5C10, F6E5, F12H4 were obtained via ELISA screening.

To test the protective efficacy of the resulting monoclonal antibodies in vivo, groups of 6–10 weeks old BALB/c mice (n = 5/group) were subcutaneous (s.c.) challenged with 600 CFU (1 MLD = 17 CFU) of *Y*. *pestis* 141. Each mouse received 100 μg of monoclonal antibody purified from murine ascites via tail vein injection 24 h before the *Y*. *pestis* challenge. Control mice (n = 5/group) received PBS. The mice were observed for 24 days after challenge. Plague was confirmed as the cause of death by plating blood samples and sections of liver, lung, spleen, and lymph node onto Congo red agar and incubating at 28℃ for 48 h to observe bacterial growth. Surviving mice were euthanized and tissues were collected to determine whether bacteria were present as above.

In the survival study, although mice died as a direct result of the experimental intervention, we used humane endpoints and euthanized mice displaying severe illness prior to the end of our experiments to minimize the pain and distress. Following challenges, the condition of mice was monitored every day. Any mice displaying severe illness as determined by weight loss of greater than 20%, a hunched posture, loss of ability to ambulate, labored breathing and ruffled fur were euthanized by CO2 inhalation. All the animal experiments completed, survivors were euthanized by CO2 exposure in accordance with IACUC policy. Death was verified by monitoring cardiac cessation and respiratory arrest. There was no unexpected death in this study. All of the survival experiments were conducted at Biosafety Laboratories of Qinghai Institute for Endemic Disease Prevention and Control.

### Humanization and expression of F2H5

The variable regions of F2H5 heavy and light chains were linked to the constant regions of human IgG1 heavy chain and kappa light chain by overlap PCR, respectively [19]. Then the heavy and light chains of the humanized antibody were cloned into pCDNA3.4 vector. Plasmids expressing the heavy and light chains were cotransfected into HEK293FT cells by lipofectin transfection. The antibodies were purified from the supernatant 48 h later by Protein G affinity chromatography. The F2H5 mutants were expressed in HEK293T cells using the TurboFect transfection reagent (Thermo Scientific) or in HEK293FT cells as above. Antibody titer in the HEK293T cells culturing supernatant was quantified by sandwich enzyme-linked immunosorbent assay (ELISA). Briefly, nunc plates (Thermo Scientific) were coated with 2 μg/mL of goat anti-human IgG overnight then blocked and washed. Supernatants were diluted in PBS and incubated on the coated plates for 1 h at 37°C. Bound antibodies were detected using an HRP-conjugated anti-human IgG antibody and 3,3’,5,5’-ltetramethylbenzidine (TMB). The reaction was stopped using 2M H_2_SO_4_ and the optical density (OD) was determined at 450 nm.

### Measuring F2H5 binding to rF1 by ELISA

Plates were coated overnight with 2 μg/mL of rF1 or F1 mutants at 4°C. The plates were blocked with 0.1% Tween 20, 5% BSA in PBS for 1 h at 37°C and then washed with 0.1% Tween 20 in PBS(PBST) at room temperature. Then serial dilutions of purified antibodies or HEK293T supernatants containing antibodies, were added to each well and incubated for 1 h at 37°C. The plates were washed and an HRP-conjugated anti-human IgG antibody was added to each well. Incubating for 1 h at 37°C, the plates were washed again and TMB was added to each well. The reaction was stopped using 2M H_2_SO_4_ and the optical density (OD) was determined at 450 nm. The half maximal effective concentration (EC_50_) was calculated by nonlinear regression (one site specific binding) in GraphPad.

### Measuring specific binding of F2H5 to rF1 by Western Blot

SDS-PAGE was performed using 120 g/L resolution gel on the Mini-PROTEIN 3 system (BIO-RAD). Briefly, a 5 μL sample was loaded into the gel and electrophoresis was performed at 80 V for 15 min followed by 180 V for 45 min. The proteins were transferred to nitrocellulose membrane using a Bio-Rad apparatus at 300 mA for 1 h. The membranes were blocked with TBST (150 mM NaCl, 0.1% Tween20, 10 mM Tris-HCl, pH8.0) containing 50 g/L nonfat milk powder for 1 h at room temperature and then washed three times with TBST. The blots were incubated with F2H5 (10 μg/mL) for 1 h at room temperature with constant agitation. The blots were washed three times with TBST and then incubated with an HRP-conjugated mouse anti-human antibody (1:10000, Sigma). The blots were washed four times with TBST and the bound peroxidase was visualized using chemiluminescence (Pierce SuperSignal Kit).

### Peptide-based ELISA

A 27-peptide array that covering the F1 antigen were 17 to 19 mers with 12 amino acid overlaps. The synthesized peptides were dissolved in dimethyl sulphoxide (DMSO) at 10 μg/mL. Plates were coated with peptide overnight and then blocked with 5% nonfat milk powder diluted in PBST at room temperature for 1 h. The plates were washed with PBST and purified F2H5 was added to each well and incubated for 1 h at 37°C. The plates were washed and an HRP-conjugated anti-human IgG antibody was added to each well. After incubating for 1 h at 37°C, the plates were washed again and TMB was added to each well. The reaction was stopped using 2M H_2_SO_4_ and the optical density (OD) was determined at 450 nm.

### Computational modeling

The atomic structures of F1 were downloaded from the PDB database. Discovery Studio 4.5 was used to model the atomic structure of F2H5. The F1/F2H5 complex was modeled using ZDOCK [20]. ZRank was a scoring function to evaluate the poses output by ZDOCK in Discovery Studio.

The residue contact frequency (RCF) score was calculated using the algorithm published by Howook et al [21]. Given that the antibody framework did not react with the antigen, only atoms in the CDR loops were used to predict the interface of the antigen-antibody complex. We also used Precision-recall curve and the associated Area Under the Curve (AUC) to evaluate the results of the RCF prediction [21]. Precision and Recall were defined as:

$$Precision=\frac{TP}{TP+FP}$$

$$Recall=\frac{TP}{TP+FN}$$

Where true positives (TP) denotes the number of correctly predicted interface residues, false positives (FP) denotes the number of residues incorrectly predicted to be in the interface, and false negatives (FN) denotes the number of residues incorrectly predicted not to be in the interface. We changed the amino acid number of predicted positive as different window sizes to get the different true positive rate and false negative rate, got a prediction-recall curve, and calculated the area under the curve.

The Mutation Energy of the F2H5 mutants and the F1 mutants was calculated using Discovery Studio 4.5.

Amino acid interface fitness (AIF) was an algorithm which captured affinity-enhancing mutants and was calculated here using parameters published by Kannan et al [22].

The calculations described above were performed using the Perl module in the Discovery Studio software.

### Structural analysis of purified mutated F1

The circular dichroism (CD) of F1 samples was analyzed with a JASCO-810 spectropolarimeter (JASCO UK, Ltd., Essex, United Kingdom). For far-UV CD, samples were diluted to 0.5 mg/mL in 50 mM phosphate buffer (pH 7.4), and a cuvette with a 0.1 mm path-length was used. For far-UV CD thermal analysis, protein samples were diluted to 0.1 mg/mL and analyzed in a 1-mm capped cuvette at 210 nm over a gradient of increasing temperatures (1°C/min). The CD spectra changed very little between 5 and 30°C; thus, 30°C was used in the normalization procedure as fully folded, and 95°C was normalized as fully unfolded. All solutions were gently degassed prior to use. The data were corrected for the buffer baseline (buffer scans performed under identical conditions) and analyzed using the standard MicroCal ORIGIN V.7 software.

## Results

### In vivo screening identified three mAbs that protected against *Y*. *pestis* infection

**Fig 1:**
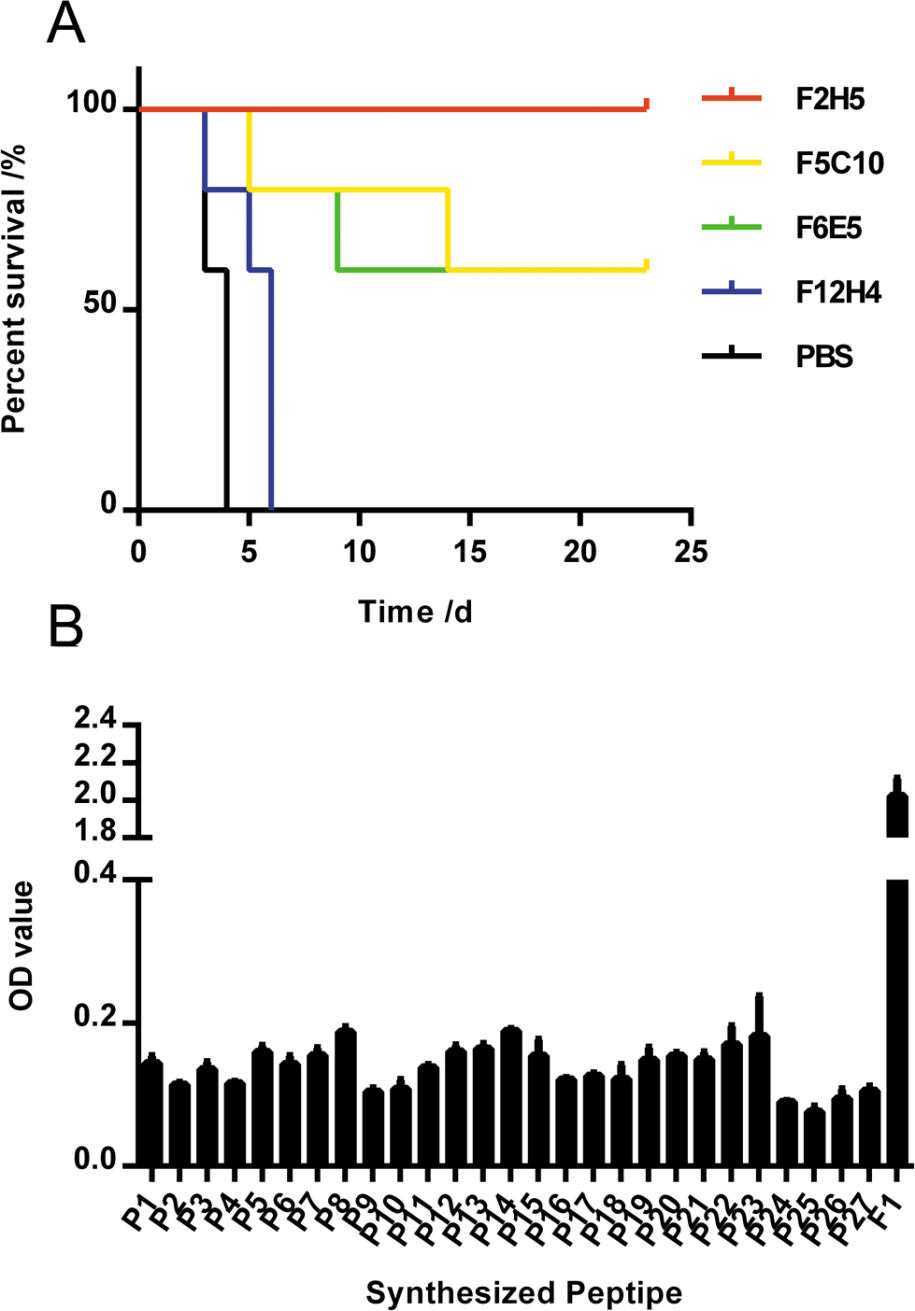
In vivo activity of monoclonal antibodies against *Y*. *pestis* and epitope analysis of F2H5 by peptide ELISA. (A) We monitored the survival of Balb/c mice (n = 5/group) who were prophylactically treated with different mAbs (100μg) intravenously 24 h prior to the challenge with *Y*. *pestis* (strain 141; 600 CFU). Survival is shown by a survival curve. (B) Peptide mapping was performed to identify a linear epitope of F2H5 using ELISA. Twenty-seven peptides covering the full-length of F1 antigen were synthesized and detected. The OD value is shown.

We screened 4 mAb lines by ELISA for reactivity to rF1. Balb/c mice were given 100 μg of antibody and 24 h later challenged with 600 CFU of *Y*. *pestis*. Three of the antibodies (F5C10 [60%]; F6E5 [60%], and F2H5 [100%]) provided some level of protection (Fig 1A).

### F2H5 recognized full length F1 but not linear peptide epitopes

To characterize the mAbs that protected against *Y*. *pestis* infection, the variable regions of F5C10, F6E5 and F2H5 genes were amplified and sequenced. Sequence alignment indicated that all three mAbs had the same heavy chain but different light chains. Given that F2H5 provided the best protection, we humanized and purified only F2H5 for further characterization in subsequent experiments [19]. Specificity of humanized F2H5 to full length F1 was demonstrated by both Western Blot and ELISA, suggesting the epitope of F2H5 may be linear. Peptide-based ELISA has been used to map linear antibody epitopes in previous studies [23,24]; therefore, 27 peptides covering the full-length of F1 were synthesized (Table 1) and detected. Unexpectedly, none of the peptides bound to F2H5 (Fig 1B).

**Table 1:** Amino acid sequences of the peptides used in mapping epitope.

NO.	AA sequence	NO.	AA sequence
P1	ADLTASTTATATLVEPA	P15	YLTFTSQDGNNHQFTTK
P2	STTATATLVEPARITLT	P16	SQDGNNHQFTTKVIGKD
P3	ATLVEPARITLTYKEGA	P17	NHQFTTKVIGKDSRDFD
P4	PARITLTYKEGAPITIM	P18	TKVIGKDSRDFDISPKV
P5	LTYKEGAPITIMDNGNI	P19	KDSRDFDISPKVNGENL
P6	GAPITIMDNGNIDTELL	P20	FDISPKVNGENLVGDDV
P7	IMDNGNIDTELLVGTLT	P21	KVNGENLVGDDVVLATG
P8	NIDTELLVGTLTLGGYK	P22	NLVGDDVVLATGSQDFF
P9	LLVGTLTLGGYKTGTTS	P23	DVVLATGSQDFFVRSIG
P10	LTLGGYKTGTTSTSVNF	P24	TGSQDFFVRSIGSKGGK
P11	YKTGTTSTSVNFTDAAG	P25	FFVRSIGSKGGKLAAGK
P12	TSTSVNFTDAAGDPMYL	P26	IGSKGGKLAAGKYTDAV
P13	NFTDAAGDPMYLTFTSQ	P27	GKLAAGKYTDAVTVTVSNQ
P14	AGDPMYLTFTSQDGNNH		

### Predicting the epitope using a bioinformatics approach

**Fig 2:**
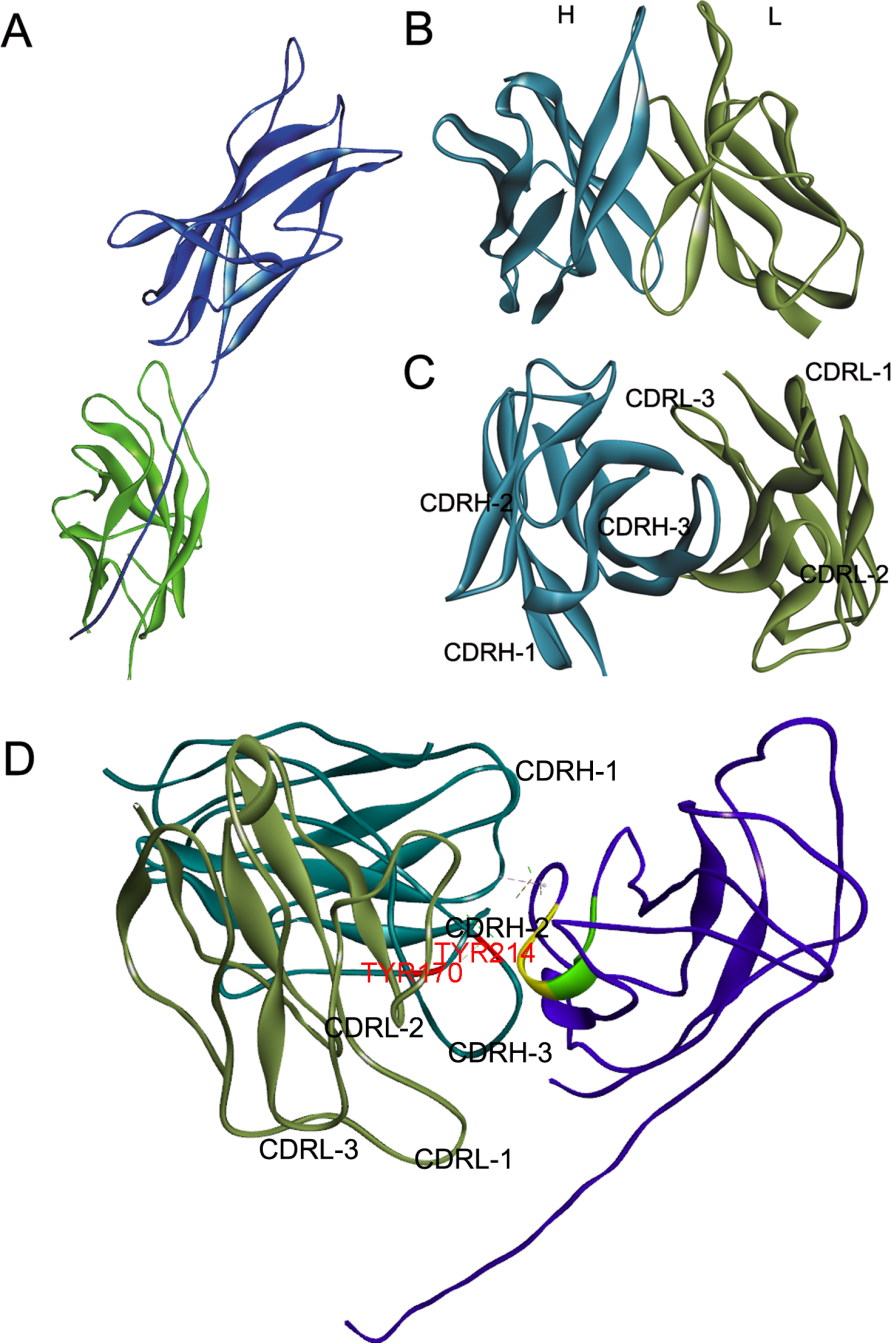
Structures of F1 (1P5U), F2H5 and F1-F2H5 complex. (A) The structure of F1 (1P5U) downloaded from the PDB database. (B) and (C) The structure of F2H5 (brown = L chain; cyan = H chain) modeled by Discovery Studio 4.5. (D) The structure of F1 (purple) and F2H5 (brown = L chain; cyan = H chain) complex filtered by the critical amino acids G^104^E^105^N^106^ and optimized by Discovery Studio 4.5. In this conformation, the L chain of F2H5 does not interact with F1, but the 3 CDR loops in the H chain do bind to F1. Within F1, residue 99–103 is colored green and residue 104–106 is colored yellow, respectively. Yellow region contains the epitope G^104^E^105^N^106^ of F2H5. The critical residues Y170 in CDRH2 and Y214 in CDRH3 of F2H5 are labeled red.

Molecular docking algorithms, such as ZDOCK and HADDOCK, that predicted macromolecule complex have been used to understand antigen-antibody interactions [20,25]. These algorithms operate by generating thousands of conformations and then scoring each conformation. Discovery Studio (Version 4.5) was used to predict atomic structure of F1/F2H5 complex using F1 structures downloaded from the PDB database and a model of F2H5 structure constructed by Discovery Studio. Five F1 structures were used to dock respectively. Three of them, 1P5U (Fig 2A), 1Z9S and 3DPB were monomers and the other two, 3DOS and 3DSN, were dimers. Schematic model of F2H5 structure is shown in Fig 2B and 2C. All five atomic structures of F1 were used to predict atomic structures of possible F1/F2H5 complexes using ZDOCK. Each ZDOCK output 2000 conformations with the highest ZRANK score. But it is a problem to distinguish which conformations is reliable.

**Fig 3:**
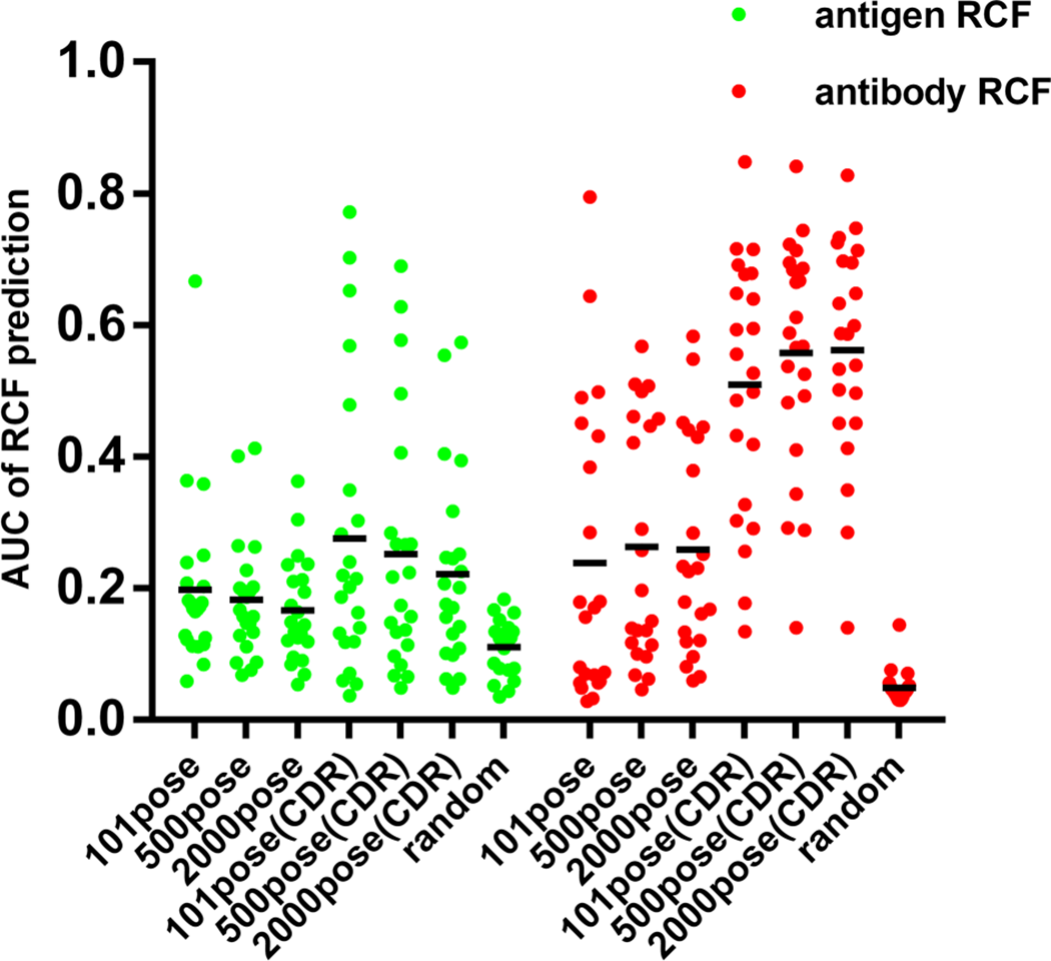
Area under the curve of RCF predictions. Base on both the whole antibody and CDR loops, the RCF scores of 22 antigen-antibody pairs were calculated using the top 101, 500, and 2000 conformations respectively. Then the areas under the PR curves were calculated and shown. AUC of random predictions were used to assess whether RCF results are better than random. Each point indicates a different antibody/antigen pairing.

Howook et al developed a method called Residue Contact Frequency (RCF) to predict the interface residues by combining protein-protein ZDOCK results [21]. RCF reflects how often a residue is present in the binding interface in a set of predicted protein-protein complex structures. Given that CDR regions of the antibody determine most interactions with antigens, we limited our RCF analysis to these regions. We choose 22 antigen-antibody pairs from Docking Benchmark 5 Database to verify our RCF analyses [26]. We also compared the effects of using different numbers of conformations to calculate RCF (Fig 3). Just like our anticipation, the accuracy of RCF prediction increased when the CDR limitation was considered. Top 101 poses, 500 poses and 2000 poses with the highest ZRANK score were used to calculate RCF, and the results were not significantly different.

**Fig 4:**
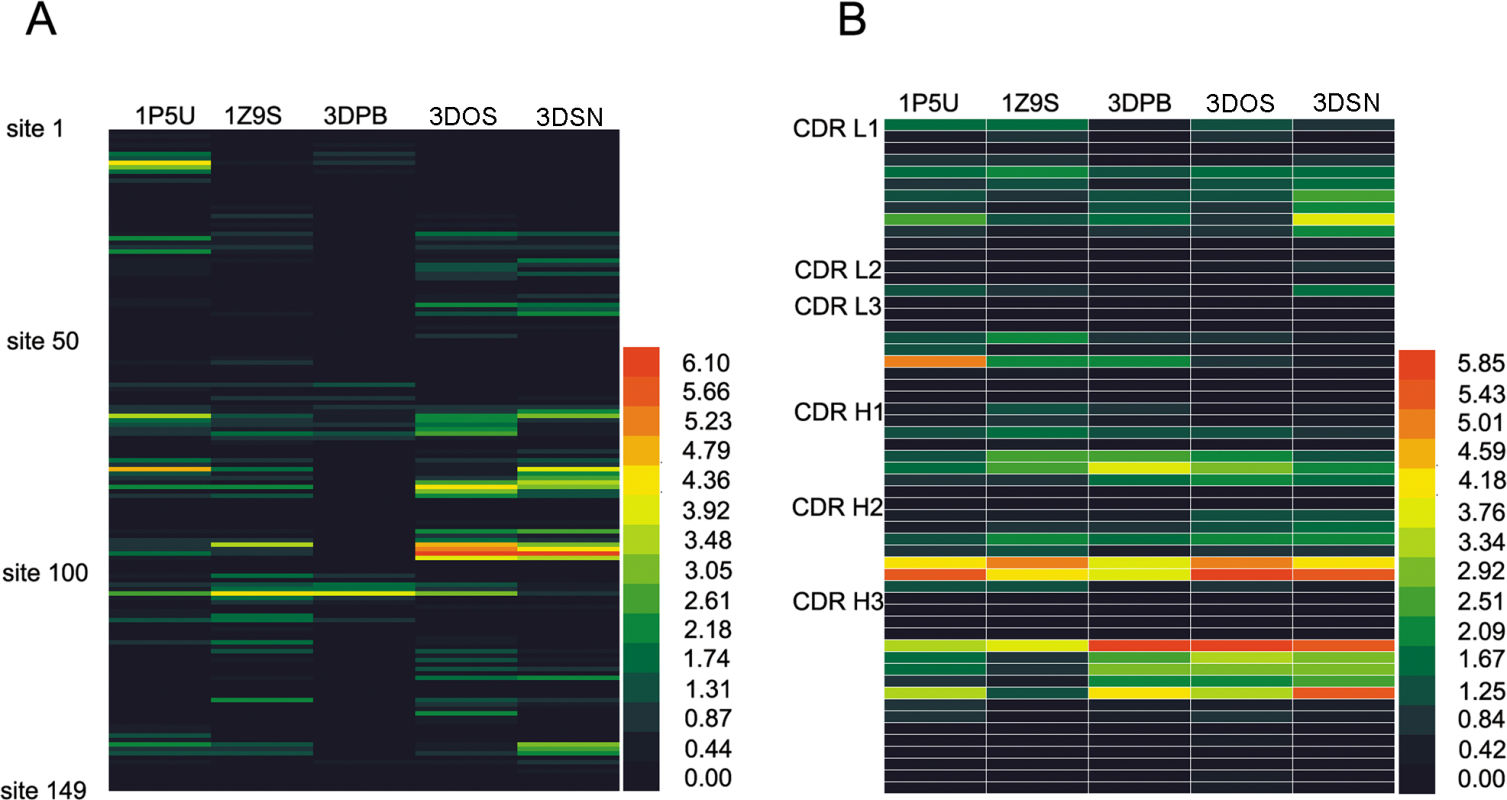
The RCF scores of F1 and F2H5. The RCF scores of F1 (A) and F2H5 (B) were calculated and shown in heat maps.

Therefore, we used the top 101 conformations with the highest ZRANK score to calculate RCF of F1 and F2H5 based on the CDR limitation to reduce the computations. The RCF calculated by five different structures of F1 is shown in a heat map in Fig 4. The top 15 residues with the highest RCF score of F1 are shown in Table 2. RCF predicted that E105 was a critical residue for F1 and F2H5 binding in all of the three monomer structures. In the dimer structures, F96 was predicted to be important for F2H5 binding.

**Table 2:** Fifteen residues of F1 with highest RCF score.

1P5U		1Z9S		3DPB		3DOS		3DSN	
Residue	RCF	Residue	RCF	Residue	RCF	Residue	RCF	Residue	RCF
GLN77	5.205	GLU105	4.418	GLU105	4.077	PHE96	6.530	PHE96	6.414
THR8	4.505	ARG94	3.547	ASN103	2.031	ASP95	5.251	ASP95	4.763
ALA65	3.499	ASN81	2.598	GLY104	1.755	ARG94	4.860	GLN77	3.936
ARG9	3.256	LYS129	2.241	PRO69	1.735	ASN81	4.685	ASP97	3.855
GLU105	2.634	ASP111	2.141	THR58	1.307	ASP97	4.004	ASN80	3.783
PRO28	2.458	THR116	2.048	THR63	1.255	GLU105	3.28	ALA65	3.261
THR139	2.452	ASP110	1.952	ASP111	1.239	HIS82	3.068	ASN81	3.237
GLU25	2.426	LYS101	1.945	GLY67	1.123	PRO69	2.656	THR139	3.108
ASN81	2.277	PRO69	1.942	THR8	1.097	ASN80	2.638	ARG94	3.080
PHE96	2.002	ASN106	1.942	LYS101	1.051	GLU40	2.602	GLY79	2.731
THR75	1.923	GLN77	1.878	ASN106	1.014	LYS132	2.595	ASP140	2.719
SER6	1.872	GLY104	1.826	SER6	0.925	LYS91	2.545	LYS91	2.671
THR10	1.862	ALA65	1.678	ASN61	0.903	GLN83	2.505	ALA141	2.566
ALA66	1.802	ASN103	1.521	MET70	0.894	ALA65	2.491	ASP78	2.502
ASP78	1.514	ALA141	1.505	ALA65	0.790	ALA66	2.208	ARG124	2.502

Results reflect the top 10% of the RCF hits; total returned was 149.

### Verifying the epitope predicted by RCF

**Fig 5:**
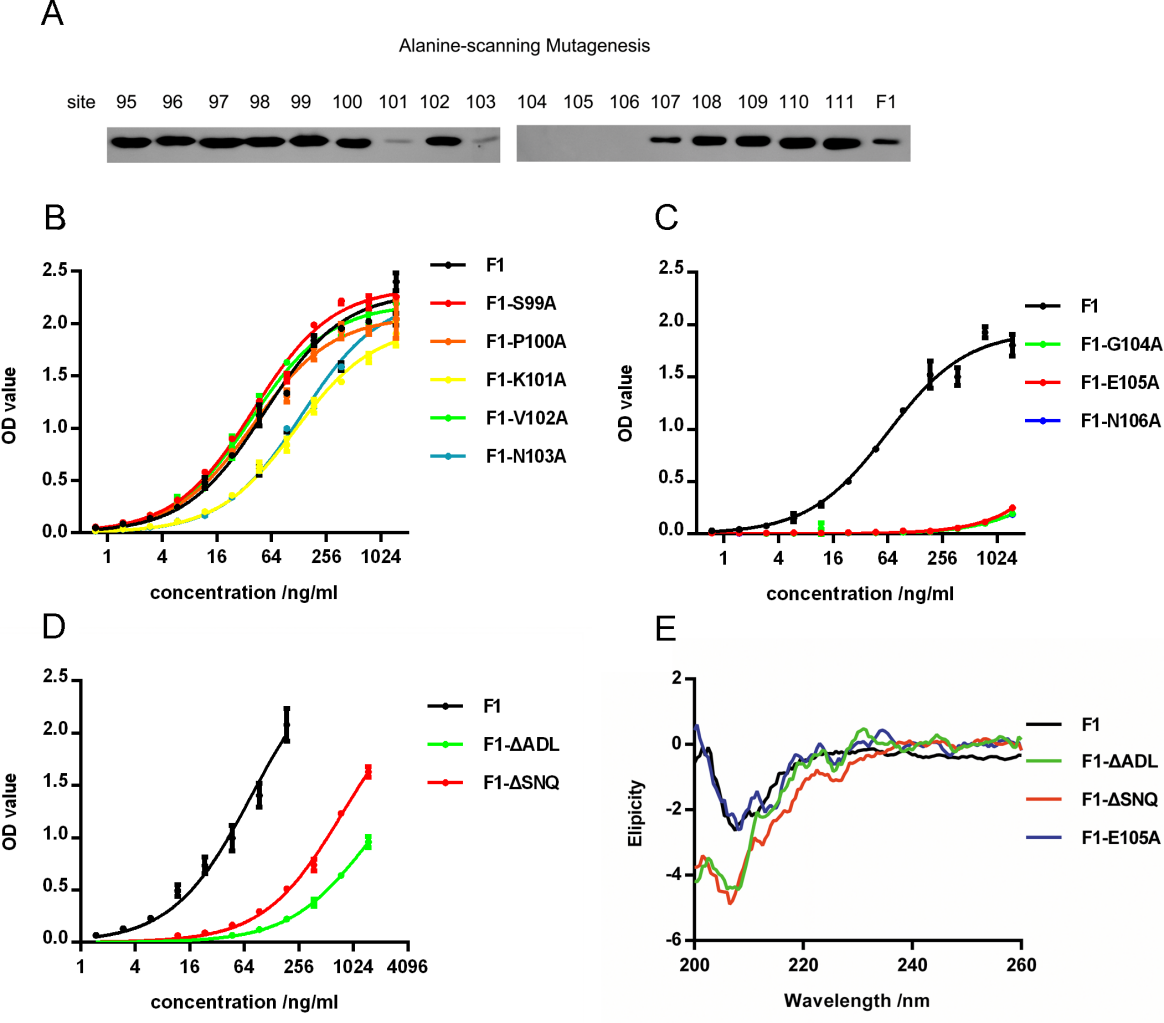
F1 mutants binding to F2H5 and analyzing the structure of F1 mutants. F1 mutants binding were determined by Western Blot (A) and ELISA (B, C and D). (E) Circular dichroism of F1 and its mutants.

To corroborate the RCF results, the binding site of F2H5 was mapped by alanine-scanning mutagenesis. F1 mutants that altered the amino acids around E105 and F96 were constructed. Residues 95–111 was mutated to alanine respectively. In both Western Blot (Fig 5A) and ELISA (Fig 5B and 5C), three mutants (F1-G104A, F1-E105A, F1-N106A) did not bind to F2H5, and two mutants (F1-K101A and F1-N103A) bound to F2H5 weakly. These results confirmed that G^104^, E^105^, and N^106^ were critical to F1/F2H5 binding.

### Characterizing the effects of mutation on the secondary structure of F1

While testing the ability of F1 mutants to bind to F2H5, we found that two mutants F1-ΔADL and F1-ΔSNQ that lacked the first three amino acids at the N terminus (F1-ΔADL) or the C terminus (F1-ΔSNQ) partially lost affinity (Fig 5D). This was unexpected as the N terminus, the C terminus and G^104^E^105^N^106^ regions are spatially distinct and F2H5 could not interact with all the three regions simultaneously. Based on the structure of the F1 dimers and the propensity of F1 to aggregate, the N and C termini appeared to be important to aggregation. Therefore, we assumed that the absence of the first three amino acids at either terminus led to structural modifications that decreased the affinity.

The secondary structures of the mutants were determined by CD. F1-ΔADL and F1-ΔSNQ were noticeably different from the native F1 on the secondary structure. However, the secondary structure of F1-E105A was comparable to native F1. (Fig 5E). This confirmed that the structure of F1-E105A mutant was analogous to native F1. But, that alterations to N or C terminus led to major structural rearrangements.

### Characterizing F2H5/F1 complex

To better understand how F2H5 was binding to F1, we filtered the predicted conformations by the epitope identified above. Then the conformation of F2H5/F1 with the highest ZRANK score among the remaining conformations was selected as the atom structure of F2H5/F1 complex. The conformation selected is shown in Fig 2D. In this conformation, we also found that residues 104–106 (Fig 2D indicated in yellow) were located in the center of the interface. Residues 99–103 (Fig 2D indicated in green) were behind residues 104–106 and hidden inside of the F1 protein. Combining with the experimental results of F1 mutants (Fig 5), it indicted that residues 104–106 was the critical amino acids, but residues 101 and 103 influenced the interaction indirectly.

Base on this conformation, we calculated the mutation energy of mutating residues 95–111 to alanine (Table 3). With the exceptions of D^110^ and D^111^, the mutation energy of the other residues coincided with the mutation experiment, that G104A, E105A and N106A had high mutation energy.

**Table 3:** The mutation energy of F1 mutations predicted in silico.

Mutations	Mutation energy /KJ/mol
ASP95>ALA	0.19
PHE96>ALA	0.19
ASP97>ALA	0.27
ILE98>ALA	0.05
SER99>ALA	0.03
PRO100>ALA	0.04
LYS101>ALA	0.15
VAL102>ALA	0.02
ASN103>ALA	0.05
GLY104>ALA	0.93
GLU105>ALA	1.19
ASN106>ALA	0.63
LEU107>ALA	0.57
VAL108>ALA	0.2
GLY109>ALA	0.12
ASP110>ALA	0.68
ASP111>ALA	1.29

In this conformation, the H chain of F2H5 was the only part recognizing F1 of the antibody. This was consistent with the variable region sequence alignment., whose results were that all the three protective mAbs have the same heavy chain.

### Predicting paratope and capturing affinity-enhance mutants of F2H5

The RCF value of each residue in the F2H5 CDR was calculated with the five F1 structures and shown in a heat map (Fig 3B). The 6 residues with the highest RCF value are shown in Table 4. Based on these results, residue 170 of CDR H2 and residue 214 of CDR H3 were predicted to interact with F1.

**Table 4:** The six residues of the F2H5 CDR with the highest RCF score.

1P5U		1Z9S		3DPB		3DOS		3DSN	
Residue	RCF	Residue	RCF	Residue	RCF	Residue	RCF	Residue	RCF
TYR170	5.637	LEU169	5.361	TYR214	6.160	TYR214	6.264	ASP218	5.728
TYR100	5.087	TYR170	4.368	ASP218	4.499	TYR170	6.123	TYR214	5.688
LEU169	4.331	TYR214	3.828	LEU169	4.037	LEU169	5.119	TYR170	5.486
ASP218	3.647	SER144	2.769	TYR170	3.856	ASP218	3.645	LEU169	4.185
TYR214	3.542	SER143	2.524	SER144	3.811	PHE215	3.409	GLN35	3.899
GLN35	2.701	GLY167	2.441	GLY216	2.992	GLY216	3.052	PHE215	3.064

Results reflect the top 10% of the RCF hits; total returned was 57.

Amino acid interface fitness (AIF) was then used to analysis the conformation of F1-F2H5 complex [22]. AIF score reflects the fitness of an amino acid at the binding site. In the conformation we selected, amino acids from the CDRH1 (SER144), CDRH2 (LEU169 and TYR170), and CDRH3 (TYR214 GLY216 and ASP218) loops interacted directly with F1. TYR214 (7.4) and TYR170 (4.3) had high AIF values. However, none of the other amino acids had a higher AIF score than tyrosine did at these two sites (Table 5). This demonstrated that these two sites were important to F1 binding and that tyrosine was the fittest amino acid for these positions.

**Table 5:** Mutations predicted to enhance the binding affinity of F2H5 using AIF.

Chain & CDR	Position	AIF	Mutations
CDRH-1	SER144	1.64	LYS, TYR, ARG
CDRH-2	LEU169	0.65	ASP, PRO, LYS, ILE, TRP, CYS, GLY, PHE, GLN, SER, ASN, VAL, TYR, GLU, ARG, THR, ALA, HIS
CDRH-2	TYR170	4.34	-
CDRH-3	TYR214	7.40	-
CDRH-3	GLY216	1.61	TRP, CYS, PHE, ASN, TYR
CDRH-3	ASP218	1.89	TRP, TYR

We also calculated the mutation energy of CDRH2 and CDRH3 site-saturation mutations base on this conformation. We hypothesized that the affinities of F2H5 and its mutants would be indistinguishable when the absolute value of Mutation Energy was less than 1 KJ/mol. Almost every mutation at TYR214 and TYR170 had positive mutation energy or resulted in negative mutation energy near 0, except for Y170R (S1 Table).

**Fig 6:**
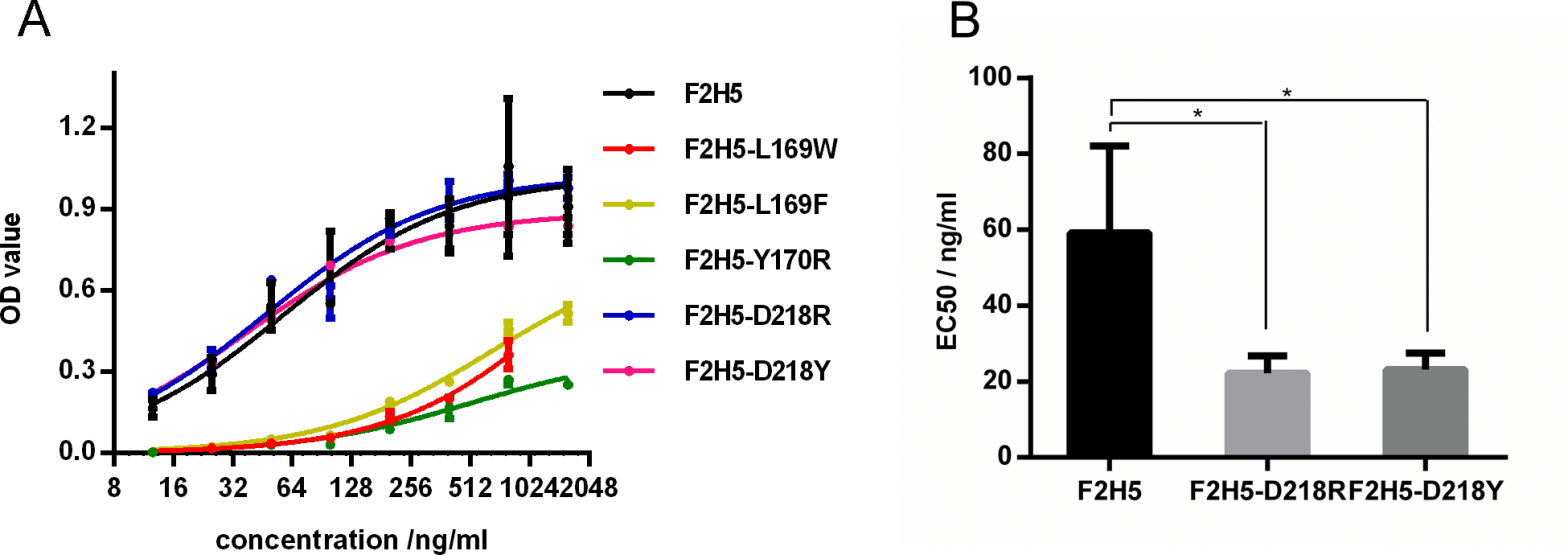
Specific binding between F2H5 mutants and F1 determined by ELISA. (A) Native and mutant F2H5 antibodies were expressed in 293T cells and detected to get their binding ability by ELISA. (B) Using purified antibodies, the EC_50_ of native F2H5, F2H5-D218R and F2H5-D218Y mutants were determined accurately. Statistics analysis was performed using one-way ANOVA with bonferroni post-test (*: p<0.05).

To confirm the results of our computational approach, we selected 20 mutants with a high absolute Mutation Energy (Table 6). The affinities of 11 mutants were predicted to weaken and those of the others were expected to enhance. The mutants were expressed in 6-well plates and the supernatant was harvested to evaluate the affinity of each mutant by ELISA. As expected, the 11 mutants with positive mutations energy no longer bound to F1 determined by ELISA. Of the remaining nine mutants which had negative mutation energy, five bound to F1, three did not bind to F1 and one mutant was not expressed (Table 6, S2 Table and S1 Fig). Compared to F2H5, two mutants, F2H5-D218R and F2H5-D218Y, bound to F1 equally well and the three remaining mutants F2H5-L169W, F2H5-L169D and F2H5-Y170R had a weaker affinity (Fig 6A). These two mutants were purified for further quantitative analysis. F2H5-D218R (EC_50_ was 22 ng/mL) and F2H5-D218Y (EC_50_ was 23 ng/mL) mutants had a lower EC_50_ than F2H5 did (EC_50_ was 59 ng/mL) (Fig 6B).

**Table 6:** Predicted mutation energy and binding results of F2H5 mutants.

Mutants	Mutation Energy/ KJ/mol	binding to F1
G166W	7.79	-
G166E	5.77	-
G167R	18.48	-
G167E	12.88	-
G168K	-1.61	-
G168R	-1.14	unexpressed
L169E	1.5	-
L169D	1.46	-
L169W	-1.51	+
L169F	-1.14	+
Y170D	1.2	-
Y170E	1.03	-
Y170R	-1.57	+
Y214P	3.93	-
Y214E	3.11	-
F215P	1.17	-
G216W	-2.51	-
G216F	-2.3	-
D218R	-1.73	+
D218Y	-1.72	+

## Discussion

We describe a novel murine antibody isolated from hybridomas, F2H5, that provides complete protection against *Y*. *pestis* infection in Balb/c mice when administered prophylactically. To alleviate immunogenicity concerns, F2H5 was humanized for developments as a novel therapeutic for humans. Western blot analysis suggested F2H5 recognized a linear epitope in the *Y*. *pestis* F1 protein. However, peptide mapping did not identify a linear epitope, suggesting a conformational one. Therefore, we used a computational approach to model the interaction between F2H5 and F1 to identify the binding site complimented by experimental approaches using targeted mutations. Given that the current computational methods cannot predict the antigen-antibody binding mode or the epitope with a high degree of accuracy, we used computer modeling to indict which residues were chosen to be mutated and the results of experimental work to verify the computer simulation to improve accuracy. Our computational and experimental approaches were generally convergent, and in this way we found that residues G^104^E^105^N^106^ in F1 reacted with CDRH2 and CDRH3 in F2H5. This particular amino acid sequence (G^104^E^105^N^106^) is contained within the predicted immunodominant F1 epitope, residues 104–117 (NGENLVGDDVVLAT) [27].

In the process of mapping the F2H5/F1 binding site, we identified that mutations to residues 101 and 103 in F1 weakened the affinity binding to F2H5, which determined by Western blot and ELISA. Our modeling results indicated that in the F1 native conformation these residues are hidden behind G^104^E^105^N^106^, which may be the reason of the weaken affinity. And the results of F1-ΔADL and F1-ΔSNQ binding experiments indicted that the N terminal and C terminal were important to maintain the high structure of F1. It also indicated that the lack of correct spatial structure of the peptide leads to the failure of epitope identification by peptide ELISA.

In addition to F2H5, we also identified F5C10 and F6E5, two monoclonal antibodies that provided partial protection against *Y*. *pestis*. All three antibodies had an identical heavy chain. The conformation we identified computationally indicated that the heavy chain of the antibody plays a major role in binding to F1. There was no contact between the antibody light chain and F1, which suggested it may only stabilize the structure of the compound. Based on these results, it appears that the heavy chain is the major determinant of protection. Both RCF and AIF suggested that Y170 in CDRH2 and Y214 in CDRH3 were critical to the binding.

Based on the computational conformation, we designed 20 mutations by Mutation Energy predicted by Discovery Studio. The affinities of 11 mutants were predicted to weaken, which was consistent with the experimental result. Of the remaining nine mutants, whose affinities were expected to enhance, five mutations bound to F1, and two of them actually had a lower EC_50_ than native F2H5. All these results suggest that the paratope of F2H5 we identified is correctly and that the conformation we selected is reliable. In the conformation we selected, residues Y170 and Y214 of F2H5 were located at the core of the interface, K169, G216 and D218 were located at the periphery of the binding interface. Mutations to D218 were found to enhance the binding affinity. In contrast, AIF and mutation energy analysis showed that tyrosine was the fittest amino acid at sites Y170 and Y214. Consistent with this, mutations at these sites failed to enhance the affinity. The finding that mutations to enhance the antibody affinity occur at the periphery of the binding site is consistent with other studies [22].

A challenge of this study was to distinguish the best one out of the thousands conformations predicted by ZDOCK. Here, we improved the RCF algorithm by considering the CDR limitation. In antibodies, only the CDR loops generally react with antigen; therefore, we used only those loops to calculate the RCF values. Based on the improved RCF values, we predicted critical residues and validated these results experimentally using mutant proteins. The findings of our study supported that the combination of computational and experimental approaches provided novel insights into understanding antibody/antigen interactions.

## Supporting information

S1 Table Mutation energy of site-saturation mutations at TYR214 and TYR170. (DOCX)

S2 Table Concentration of 20 F2H5 mutants. (DOCX)

S1 Fig Binding of 20 F2H5 mutants to F1. The supernatant was harvested to evaluate the affinity of each mutant by ELISA. Purified F2H5 (2μg/mL) and the supernatant containing F2H5 were used as positive control. The supernatant containing 5F10, an antibody of Chikungunya virus, was used as negative control. Red represent the mutants predicted with enhancing affinity. Green represent the mutants predicted with weaken affinity.
(TIF)
