# Supplementary material for: Identification and characterization of a neutralizing monoclonal antibody that provides complete protection against Yersinia pestis
Source: PLoS One. 2017 May 9;12(5):e0177012. doi: 10.1371/journal.pone.0177012 (PMC5423616; doi:10.1371/journal.pone.0177012)
Supplement: S1 Table — (DOCX) [file pone.0177012.s001.docx]

S1 Table. Mutation energy of site-saturation mutations at TYR214 and TYR170

| Amino acid | Mutations energy at Y170 /KJ/mol | Mutations energy at Y214 KJ/mol |
| --- | --- | --- |
| ALA | 0.36 | 1.6 |
| ARG | -1.57 | -0.23 |
| ASN | -0.04 | 0.99 |
| ASP | 1.2 | 2.68 |
| CYS | 0.39 | 1.44 |
| GLN | 0.19 | 3.08 |
| GLU | 1.03 | 3.11 |
| GLY | 0.48 | 2.41 |
| HIS | -0.06 | 0.09 |
| ILE | 0.07 | 0.59 |
| LEU | -0.05 | 1.24 |
| LYS | -0.13 | 0.68 |
| MET | 0.43 | 1.37 |
| PHE | -0.43 | 0.05 |
| PRO | -0.04 | 3.93 |
| SER | 0.38 | 1.85 |
| THR | 0.09 | 1.5 |
| TRP | -0.27 | -0.61 |
| VAL | 0.08 | 0.49 |
