# Supplementary material for: Identification and characterization of a neutralizing monoclonal antibody that provides complete protection against Yersinia pestis
Source: PLoS One. 2017 May 9;12(5):e0177012. doi: 10.1371/journal.pone.0177012 (PMC5423616; doi:10.1371/journal.pone.0177012)
Supplement: S2 Table — (DOCX) [file pone.0177012.s002.docx]

S2 Table. Concentration of 20 F2H5 mutants.

| Mutants | Concentration/ μg/mL |
| --- | --- |
| G166W | 1.42 |
| G166E | 1.08 |
| G167R | 1.62 |
| G167E | 1.73 |
| G168K | 0.27 |
| G168R | -0.18 |
| L169E | 1.48 |
| L169D | 1.31 |
| L169W | 0.82 |
| L169F | 1.86 |
| Y170D | 1.19 |
| Y170E | 1.99 |
| Y170R | 1.59 |
| Y214P | 1.75 |
| Y214E | 2.24 |
| F215P | 1.92 |
| G216W | 1.07 |
| G216F | 0.92 |
| D218R | 1.75 |
| D218Y | 1.80 |
| F2H5 | 2.45 |
| 5F10 | 1.09 |
