# Supplementary figures and images for: Identification and characterization of a neutralizing monoclonal antibody that provides complete protection against Yersinia pestis
Source: PLoS One. 2017 May 9;12(5):e0177012. doi: 10.1371/journal.pone.0177012 (PMC5423616; doi:10.1371/journal.pone.0177012)

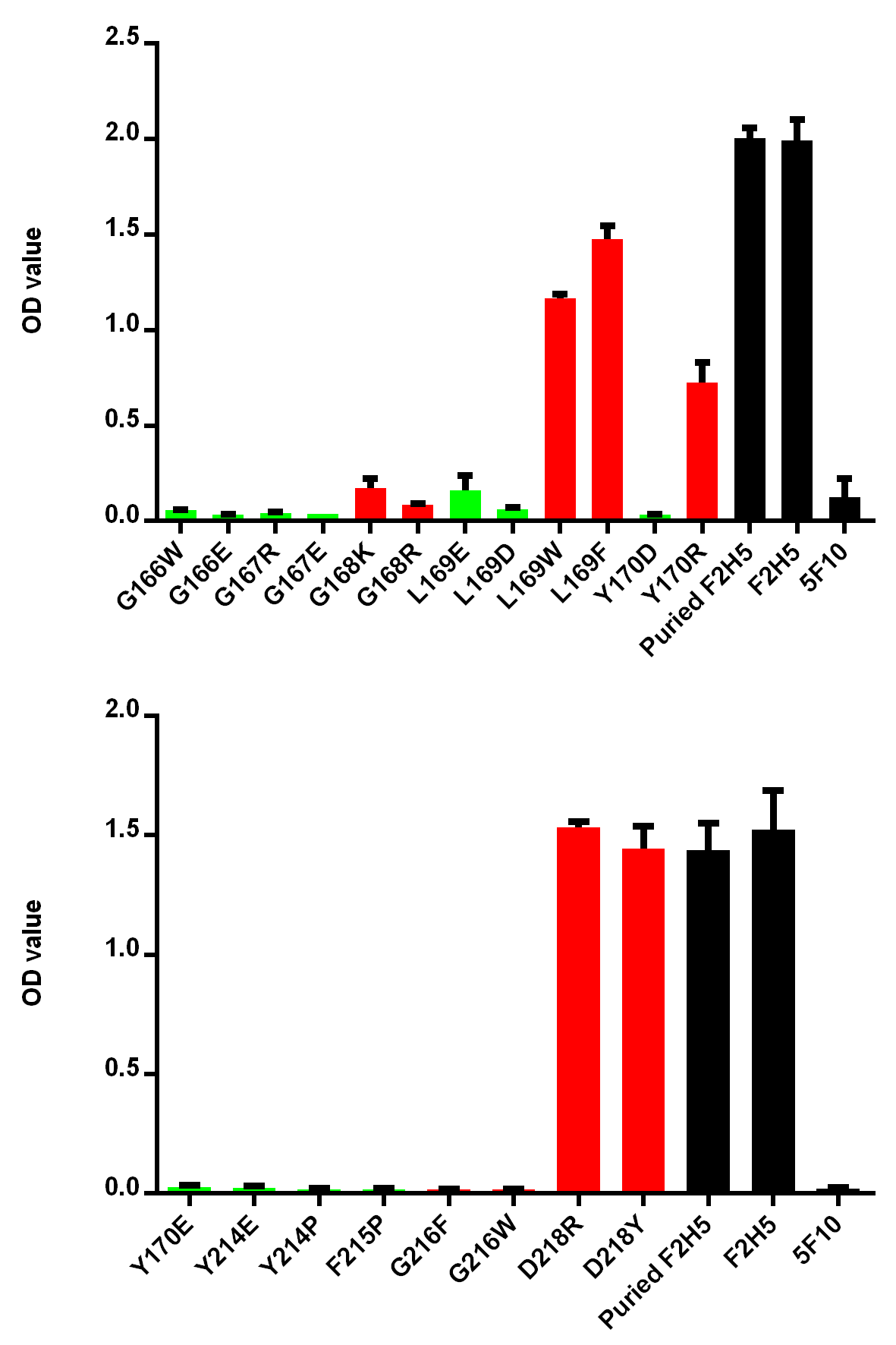

Supplement: S1 Fig — The supernatant was harvested to evaluate the affinity of each mutant by ELISA. Purified F2H5 (2μg/mL) and the supernatant containing F2H5 were used as positive control. The supernatant containing 5F10, an antibody of Chikungunya virus, was used as negative control. Red represent the mutants predicted with enhancing affinity. Green represent the mutants predicted with weaken affinity. (TIF) [file pone.0177012.s003.tif]
